# Identification and Expression Profile Analysis of the *OSCA* Gene Family Related to Abiotic and Biotic Stress Response in Cucumber

**DOI:** 10.3390/biology11081134

**Published:** 2022-07-28

**Authors:** Shuting Yang, Chuxia Zhu, Jingju Chen, Jindong Zhao, Zhaoyang Hu, Shiqiang Liu, Yong Zhou

**Affiliations:** 1 College of Bioscience and Bioengineering, Jiangxi Agricultural University, Nanchang 330045, China; shutingyang_2020@163.com (S.Y.); z18979935173@163.com (C.Z.); 13435057881@163.com (J.C.); zjd13519442751@163.com (J.Z.); huzhaoyang@jxau.edu.cn (Z.H.); 2 Key Laboratory of Crop Physiology, Ecology and Genetic Breeding, Ministry of Education, Jiangxi Agricultural University, Nanchang 330045, China

**Keywords:** cucumber, OSCA, hypertonic stress, biotic stress, expression analysis

## Abstract

**Simple Summary:**

Hyperosmolality-gated calcium-permeable channels (OSCAs) are calcium nonselective cation channel proteins involved in multiple biological processes. In this work, the members of the *OSCA* family in cucumber were systematically analyzed, including their sequence characteristics, phylogenetic relationships, conserved motifs, gene structures, promoter regions, and tissue expression patterns. In addition, the effects of different osmotic-related abiotic stresses [salt (NaCl), drought (PEG), and abscisic acid (ABA)] and three biotic stresses [powdery mildew (PM), downy mildew (DM), and root-knot nematode (RKN)] on *OSCA* family genes were also determined. The results indicated that cucumber *OSCA* genes play important roles in response to osmotic-related abiotic stresses and pathogen invasion. Overall, this study lays a foundation for research on the biological function and evolutionary process of *OSCA* family genes in cucumber.

**Abstract:**

Calcium ions are important second messengers, playing an important role in the signal transduction pathways. *Hyperosmolality gated calcium-permeable channels* (*OSCA*) gene family members play critical modulating roles in response to osmotic-related abiotic stress as well as other abiotic and biotic stresses, which has been reported in many plant species such as *Arabidopsis*, rice, maize, and wheat. However, there has been no report about the identification and expression profile of the *OSCA* genes in cucumber. In this study, a total of nine *OSCA* genes were identified, which are unevenly distributed on the six chromosomes of cucumber. Phylogenetic analysis revealed that the OSCAs of cucumber, *Arabidopsis*, rice and maize were clustered into four clades. The motif arrangement of CsOSCAs was strongly conserved, and the *CsOSCA* genes in each group had similar genetic structure. A total of 11 and 10 types of *cis*-elements related to hormone and stress, respectively, were identified in the promoter regions of *CsOSCA* genes. Gene expression analysis indicated that the *CsOSCA* genes have different expression patterns in various tissues, and some of them were regulated by three osmotic-related abiotic stresses (salt, drought and ABA) and three biotic stresses (powdery mildew, downy mildew, and root-knot nematode). As the first genome-wide identification and characterization of the *OSCA* gene family in cucumber, this study lays a foundation for research on the biological function and evolutionary process of this gene family, which is of great significance for exploiting stress resistant cucumber varieties.

## 1. Introduction

Plants have multiple signaling pathways in response to a variety of biotic and abiotic stresses, such as insects and microbial pathogens, drought, soil salinity, heavy metals, and extreme temperature, which have great impacts on the survival and productivity of plants [1,2]. As a result, plants have evolved complex defense systems to protect themselves under various adverse conditions. As an important second messenger, calcium (Ca^2+^) is involved in multiple cell signal transduction pathways in plants. Upon external environmental stimuli on plants, the concentration of cytosolic free calcium ([Ca^2+^]_cyt_) will increase rapidly (within five seconds), and a number of stress-related genes will be induced to regulate the stress response [3,4]. Plants convey various signals when exposed to different Ca^2+^ signatures, which can be effectively regulated by a series of Ca^2+^ transporters, such as glutamate receptor-like (GLR) proteins, MscS like proteins (MSLs), cyclic nucleotide-gated channels (CNGCs), two-pore cation channels (TPCs), and annexins (ANNs) [5,6,7,8,9]. Notably, hyperosmolality-gated calcium-permeable channels (OSCAs) are calcium nonselective cation channel proteins that have been proven to act as a receptor protein for hypertonic stress and in response to other types of stress stimuli in plants [10,11]. The mutation of OSCA1 (REDUCED-HYPEROSMOLALITY-INDUCED-[Ca^2+^]_cyt_-INCREASE 1) in *Arabidopsis* led to a low hyperosmolality-induced increase in [Ca^2+^]_cyt_ and impairment of osmotic calcium ion signaling in guard cells and root cells, as well as transpiration and root growth regulation [10].

OSCA proteins are distributed as multigene families in eukaryotic species possessing conserved DUF221 (Domain of Unknown Function 221) with 11 transmembrane helices and a cytosolic soluble domain that forms a homodimer of two pores [12,13,14]. Genome-wide survey and identification of the *OSCA* gene family have been widely conducted in different species, and the gene number ranged from 11 members in rice to 42 members in wheat [10,15,16,17]. In recent years, many reports have indicated the roles of *OSCA* genes in osmotic-related abiotic stress response. For example, overexpression of maize *ZmOSCA2.4* in *Arabidopsis* conferred tolerance to drought stress with increases in the expression of drought tolerance-related genes [18]. Overexpression of several rice *OsOSCA* genes in *Arabidopsis* mutant *osca1* could complement both hyperosmolality-induced and salt stress-induced increases in cytosolic Ca^2+^ [19,20]. In addition, in *Arabidopsis*, Ca^2+^ permeable OSCA1.3 and the N-terminal phosphorylation of OSCA1.3 mediated by receptor-like cytoplasmic kinase protein BIK1 are critical for stomatal closure during pathogen-associated molecular pattern (PAMP) immunity processes, while OSCA1.3 does not regulate stomatal closure induced by abscisic acid (ABA) [11]. Recently, *Arabidopsis* OSCA1.1 was found to play a crucial role in root hydrotropism by regulating the expression of MIZU-KUSSEI 1 (MIZ1), particularly at the later stage of hydrotropic response [21].

As a kind of vegetable crop with rich nutrients and crispy taste, cucumber is widely cultivated in the world. Unfortunately, it often suffers from various biotic stresses (such as powdery mildew and downy mildew) and abiotic stress (drought, cold, osmotic, and salt) under natural conditions, which seriously affect its yield and quality [22,23]. The *OSCA* family has been investigated in many plant species, such as *Arabidopsis* [10], rice [15], maize [18,24], and wheat [17], whereas the members of *OSCA* family in cucumber have not been identified. In this study, we systematically identified the *OSCA* gene family members and analyzed their sequence characteristics, promoter regions, and tissue expression patterns. In addition, the effects of different osmotic-related abiotic stresses [salt (NaCl), drought (PEG), abscisic acid (ABA)] and three biotic stresses [powdery mildew (PM), downy mildew (DM), and root-knot nematode (RKN)] on *OSCA* family genes were also examined using quantitative real-time RT-PCR (qRT-PCR) and public transcriptome datasets. Some cucumber *OSCA* genes were found to be differentially expressed in responses to various biotic stresses, which has not been reported so far in other plant species. This study lays a foundation for research on the biological function and evolutionary process of *OSCA* family genes in cucumber.

## 2. Materials and Methods

### 2.1. Identification of OSCA Family Genes in Cucumber

The genomic database of the cucumber variety ‘9930’ was downloaded from the CuGenDB (http://cucurbitgenomics.org/organism/2, accessed on 4 July 2022). According to previous research, the HMM model (Pfam accession: PF02714) from the Pfam database (http://pfam.xfam.org/, accessed on 4 July 2022) was used to search the genomic protein database of the cucumber with the HMMER3.0 software. In addition, the OSCA proteins reported in *Arabidopsis* and rice were also employed as queries to identify the homologous OSCAs in CuGenDB by performing BLASTp program [9,15]. The putative members of *OSCA* genes were checked with the complete DUF221 protein domain with the tools of SMART (http://smart.embl-heidelberg.de/, accessed on 4 July 2022) and InterProScan (http://www.ebi.ac.uk/interpro/interproscan.html/, accessed on 4 July 2022). The ProtParam online software (http://www.expasy.org/, accessed on 4 July 2022) was used to analyze the physical parameters of CsOSCA proteins, including the amino length, molecular weight (MW), isoelectric point (pI), grand average of hydropathicity (GRAVY).

### 2.2. Sequence Alignment and Phylogenetic Analysis

The online MAFFT (https://www.ebi.ac.uk/Tools/msa/mafft/, accessed on 4 July 2022) tool was used to align the full-length amino acid sequences of all OSCA proteins of cucumber, *Arabidopsis*, rice and maize with the default setting parameters. The alignment files were uploaded to MEGA7.0 software to construct a phylogenetic tree with bootstrap test performed with 1000 replications. The phylogenetic tree was created using the neighbor-joining (NJ) method.

### 2.3. Conserved Motif and Gene Structure Analysis

The Multiple Expectation Maximization for Motif Elicitation (MEME) (http://meme-suite.org/tools/meme, accessed on 4 July 2022) was used to analyze the conserved motifs of CsOSCA proteins, with all parameters being set as default but the maximum number of different motifs was set as 10. The annotation information and genomic sequence of cucumber were used to draw the exon and intron structures of the *CsOSCA* genes with Gene Structure Displayer server (GSDS) [25]. Then, the TBtools software was used for visual analysis [26].

### 2.4. Chromosomal Location and Promoter Analysis

The positional information about starting and ending points of each *CsOSCA* gene was obtained from CuGenDB, and their chromosomal locations were displayed with the MapInspect software. To analyze the *cis*-acting elements of the promoter regions of *CsOSCA* genes, 2.0-kb upstream promoter sequences of the start codon (ATG) of *CsOSCA* genes were acquired, and the diverse hormone- and stress-responsive *cis*-elements were derived with the PlantCARE server (http://bioinformatics.psb.ugent.be/webtools/plantcare/html/, accessed on 4 July 2022).

### 2.5. In Silico Expression Analysis Based on RNA-Seq

For expression analysis of *CsOSCA* genes in different tissues of cucumber, the RNA-seq data of different tissues such as roots, stems, leaves, male and female flowers, ovaries, and tendrils were obtained from the NCBI SRA database with the data code of PRJNA80169. In order to explore the expression patterns of the *CsOSCA* genes in response to various abiotic and biotic stresses, the public transcriptome datasets, including powdery mildew (PM, BioProject ID: PRJNA321023), downy mildew (DM, BioProject ID: PRJNA285071), and root-knot nematode (RKN, accession ID: SRP125669), were downloaded from the NCBI SRA database. The relative gene expression of *CsOSCA* genes was represented as transcripts per million reads (TPM) according to our previous study [2], and the heatmaps were drawn with the TBtools software based on the normalized log_2_ (TPM + 1) values. Genes up- or down-regulated by two-fold or more (treatment/control) were designated as differentially expressed genes (DEGs).

### 2.6. Plant Materials and Treatments

The cucumber variety ‘9930’ was used in this study. Two week-old cucumber seedlings grown in hydroponic culture were treated with either 10% polyethylene glycerol-6000 (PEG) or 200 mM NaCl or 100 μM ABA solution as described previously [22], and the leaves were sampled at four time points (0, 6, 12, and 24 h) after treatment, among which the samples at 0 h were set as non-treated control. Three biological repetitions were performed, and all samples were stored individually at −80 °C.

### 2.7. RNA Extraction, cDNA Synthesis, and qRT-PCR

Total RNA from the collected leaf samples was isolated using RNA-easy^TM^ Isolation Reagent Vazyme cat (Vazyme, Nanjing, China) according to the manufacturer’s instructions. About 5 μg of RNA was reverse transcribed as single-stranded cDNA with HiScript^®^Ⅱ Q RT SuperMix for qPCR (+gDNA wipe) (Vazyme, Nanjing, China). Quantitative real-time RT-PCR (qRT-PCR) was performed in triplicate on the Roche Lightcyler 480II PCR System using the 2× ChamQ Universal SYBR qPCR Master Mix (Vazyme, Nanjing, China). The amplification conditions were 95 °C for 30 s, followed by 40 cycles of 95 °C for 10 s and 60 °C for 30 s. The correlative expression of *CsOSCA* genes was calculated with the 2^−ΔΔCt^ method using the housekeeping gene *ACTIN* as an internal reference [22]. Statistical analysis was performed using the statistical product and service solution (SPSS) software by one-way ANOVA and Tukey-HSD test, where *p* < 0.05 indicates significant difference. The gene-specific primers for qRT-PCR are listed in Table S1.

## 3. Results

### 3.1. Identification and Characterization of OSCA Family Genes in Cucumber

A total of nine *CsOSCA* genes with the conserved DUF221 domain were identified using the HMMER3.0 software by searching the cucumber genome, which were designated as *CsOSCA1.1*–*CsOSCA4.1* based on their homology with *Arabidopsis OSCA* genes. The gDNA length of *CsOSCA* genes ranged from 3079 bp (*CsOSCA4.1*) to 9733 bp (*CsOSCA1.1*), and the CDS length ranged from 2136 bp (*CsOSCA2.3*) to 2430 bp (*CsOSCA4.1*). The CsOSCA proteins contained 712 aa (CsOSCA2.2) to 809 aa (CsOSCA4.1), and their molecular weight ranged from 80.66 kDa (CsOSCA2.3) to 92.01 kDa (CsOSCA4.1). Most CsOSCA proteins (8 out of 9) were alkaline. The GRAVY coefficient was higher than 0, suggesting that they are hydrophobic proteins (Table 1).

### 3.2. Phylogenetic Analysis of OSCA Proteins

**Figure 1:**
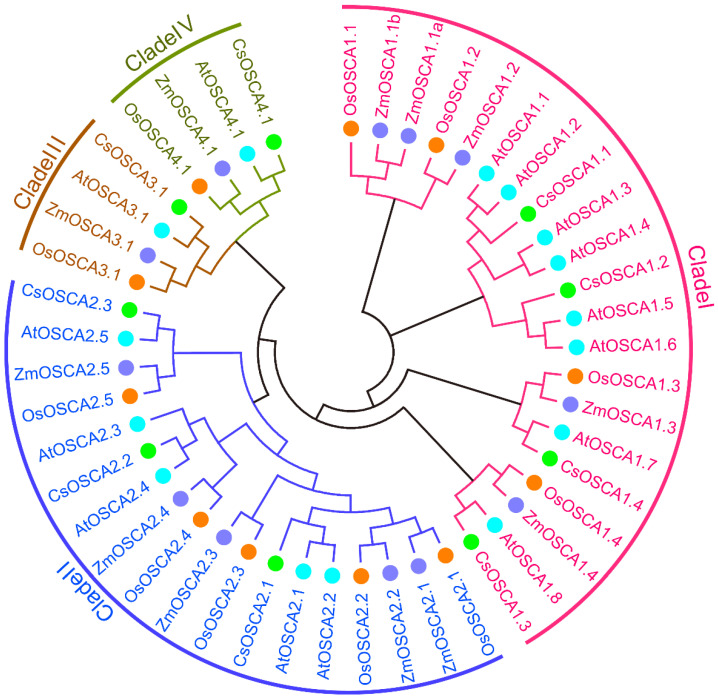
Phylogenetic relationships of OSCA proteins in cucumber, *Arabidopsis*, rice and maize.

To uncover the phylogenetic relationships of the OSCA protein family in cucumber and other plants, a phylogenetic analysis of 47 OSCA proteins from cucumber, *Arabidopsis*, rice and maize was performed based on their full-length amino acid sequences (Figure 1). These OSCA proteins were divided into four clades (Clade I–IV). Clade I and II covered the most CsOSCA numbers (four and three, respectively), and both Clade III and IV had only one OSCA member among the four species (Figure 1). Notably, each clade comprised OSCA members from the four plant species (Figure 1), indicating that the *OSCA* gene family has undergone species-specific expansion during evolution.

### 3.3. Comparison of Conserved Motifs and Gene Structures of CsOSCAs

**Figure 2:**
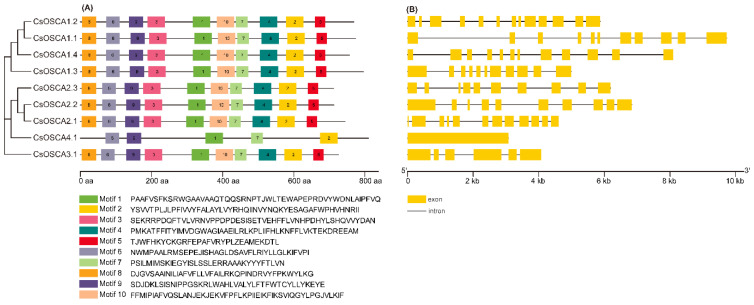
Conserved motif arrangement (**A**) and gene structure diagram (**B**) of *OSCA* gene family in cucumber based on the phylogenetic relationship. (**A**) Distribution of conserved motifs in CsOSCA proteins identified by MEME. The length and name of conserved motifs are represented by boxes with different colors and their sequences are indicated below. (**B**) Exon-intron structure of *CsOSCA* genes. The yellow boxes and black lines represent exons and introns, respectively.

To better analyze the conserved structure of CsOSCA proteins, the MEME online tool and TBtools software were used to draw the motif distribution. As shown in Figure 2A, a total of 10 conserved motifs were identified, and all CsOSCAs shared the same conserved motifs and arrangement, expect for CsOSCA4.1, which was lack of motif 3, motif 4, motif 5, and motif 10. Gene structure analysis demonstrated that the *CsOSCA* genes in Clade I and II possessed 9–12 introns; *CsOSCA3.1* had five introns; while *CsOSCA4.1* was intronless (Figure 2B).

### 3.4. Chromosomal Location of CsOSCA Genes

**Figure 3:**
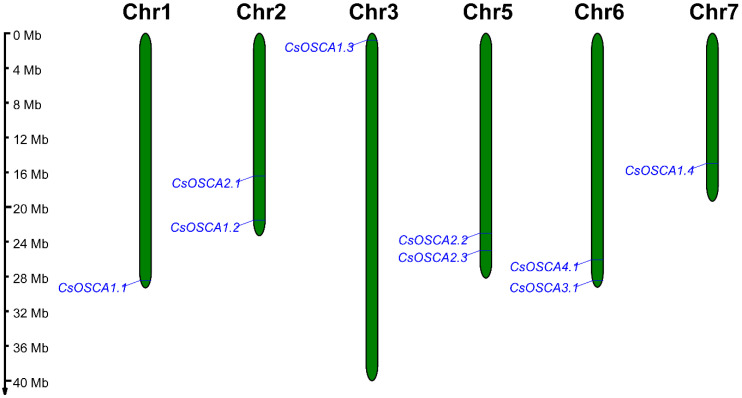
Distribution of the *CsOSCA* genes on six cucumber chromosomes. The scale on the left represents megabases (Mb).

The location of *CsOSCA* genes on each chromosome was determined based on the genomic annotation downloaded from CuGenDB. As shown in Figure 3, nine *CsOSCA* genes were unevenly located on six cucumber chromosomes. Chromosome 1, 3 and 7 each contained one *CsOSCA* gene, and chromosome 2, 5 and 6 each possessed two *CsOSCA* genes.

### 3.5. Cis-Elements in the Promoters of CsOSCA Genes

**Figure 4:**
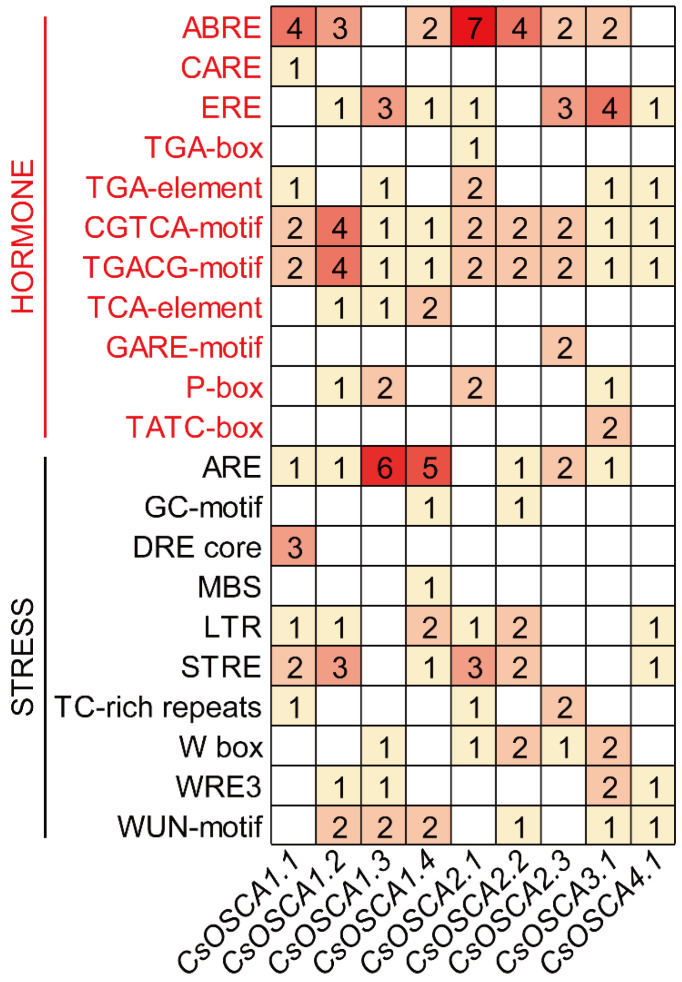
Distribution of hormone- and stress-related *cis*-elements in *CsOSCA* promoters.

To investigate the potential biological functions of *CsOSCA* genes, the promoter region of *OSCA* genes was extracted and analyzed by PlantCARE server. As a result, a total of 11 and 10 types of *cis*-acting elements related to hormone and stress response were screened out, respectively (Figure 4). Amongst the hormone-related *cis*-elements, ARBE (involved in ABA response) distributed in most of *CsOSCA* genes (except for *CsOSCA1.3* and *CsOSCA4.1*) and with *CsOSCA2.1* owning the largest number, indicating that *CsOSCA2.1* might play an important role in ABA response. There were 14 ERE *cis*-elements (related to ethylene reaction) unevenly distributed among seven *CsOSCA* genes, suggesting that the *CsOSCA* genes might play a critical role in fruit ripening. In addition, seven, four and 10 elements were filtered out in the *CsOSCA* genes in response to auxin (including TGA-box and TGA-element), salicylic acid (SA, including TCA-element) and gibberellin (GA, including GARE-motif, P-box and TATA-box), respectively. MeJA-related *cis*-acting elements (including CGTCA-motif and TGACG-motif) were present in all *CsOSCA* genes, particularly in *CsOSCA1.2*. Among the elements related to abiotic stress, a total of 19 *cis*-element related to anaerobic induction (including ARE and GC-motif) were distributed on seven *CsOSCA* genes, and it is worth noting that both *CsOSCA1.3* and *CsOSCA1.4* had six *cis*-elements responsive to anaerobic stress, suggesting that these two genes are important in adaptation to hypoxia stress. Additionally, three DRE core (involved in drought response) elements and one MBS (drought-related response) element were present in *CsOSCA1.1* and *CsOSCA1.4*, respectively, suggesting that they have important functions in response to drought stress. LTR (low-temperature responsiveness) elements and STRE (stress-response element) elements were present in the same six *CsOSCA* genes, and there were nine WUN-motifs (wound-responsive element) in six *CsOSCA* genes, indicating that the *CsOSCA* genes are important in abiotic stress response (Figure 4).

### 3.6. Tissue Expression Profiles of CsOSCA Genes

**Figure 5:**
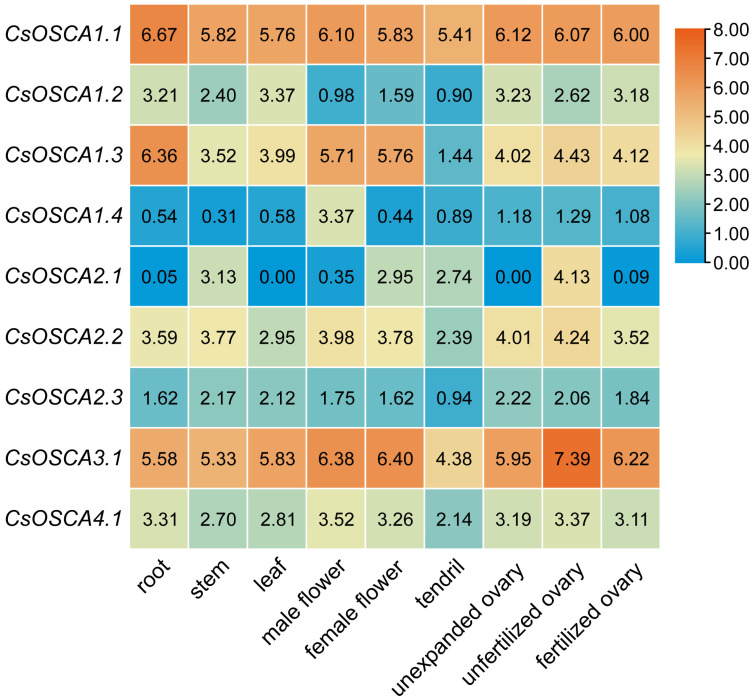
Expression profiles of *CsOSCA* genes in different cucumber tissues.

The gene-specific expression of *CsOSCA* genes in roots, stems, leaves, male and female flowers, ovaries, and tendrils was examined based on the RNA-seq data in a previous study [27]. Notably, *CsOSCA1.1*, *CsOSCA1.3*, *CsOSCA2.2*, *CsOSCA3.1* and *CsOSCA4.1* exhibited constitutive expression in all tested tissues, while other *CsOSCA* genes were differentially expressed in different tissues (Figure 5). For example, *CsOSCA2.1*, *CsOSCA2.2* and *CsOSCA3.1* showed a higher transcriptional level in unfertilized ovaries than in other tissues, while *CsOSCA1.3* had a relatively higher expression level in the root. Additionally, *CsOSCA1.4* was preferentially expressed in the male flower, implying its possible role in male flower development. Moreover, *CsOSCA2.1* had specific expression in unfertilized ovaries but not in unexpanded or fertilized ovaries, suggesting that it may play a specific role in ovary development (Figure 5).

### 3.7. Expression Patterns of the CsOSCA Genes under Abiotic Stress and ABA Treatment

**Figure 6:**
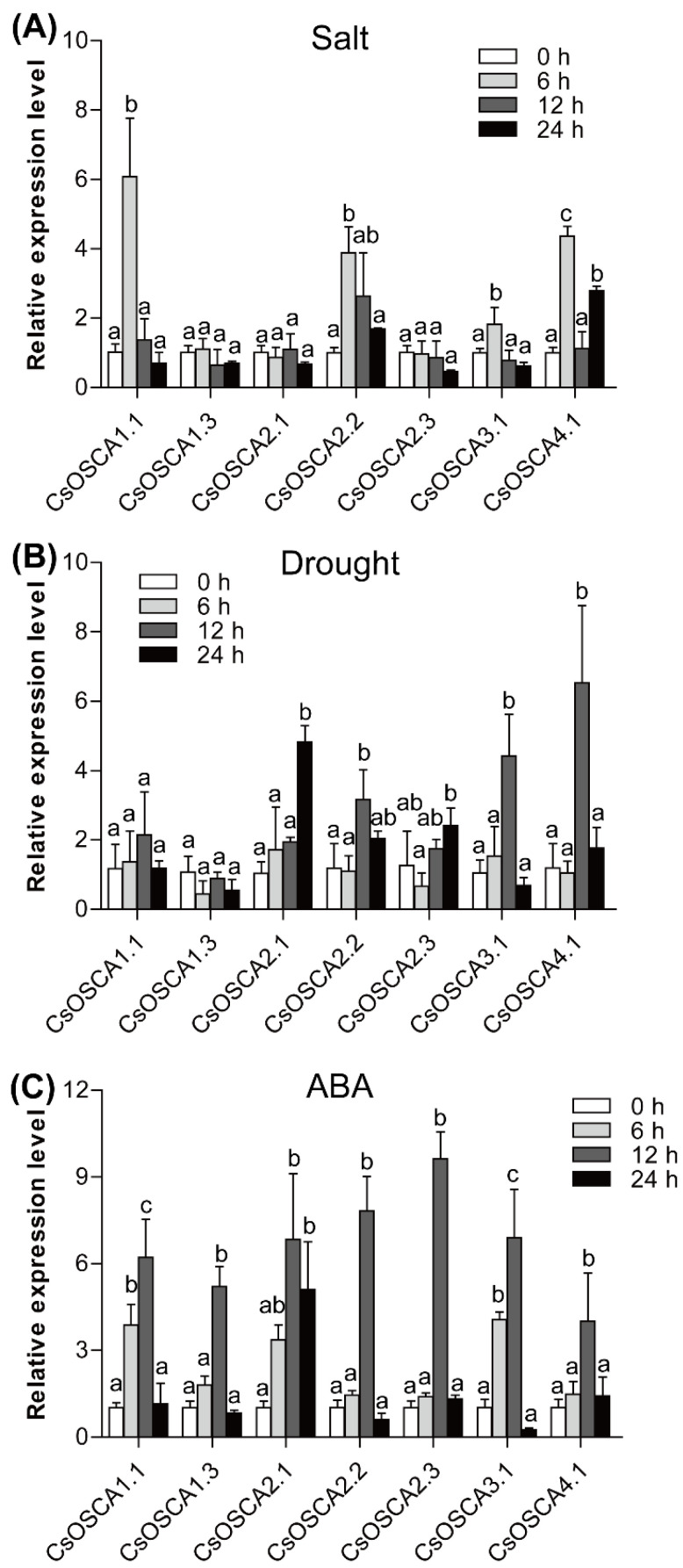
Relative expression analysis of seven *CsOSCA* genes under salt (**A**), drought (**B**), and ABA (**C**) treatments based on qRT-PCR. Error bars represent standard deviation (SD). Different lowercase letters indicate statistically significant variations in expression at *p* <  0.05.

To analyze the expression patterns of *CsOSCA* genes in response to abiotic stress, we selected seven *CsOSCA* genes for qRT-PCR analysis under salt, drought, and ABA stress conditions. Under salt stress, *CsOSCA1.1*, *CsOSCA2.2*, *CsOSCA3.1* and *CsOSCA4.1* exhibited remarkable increases in transcription level relative to the control, and their expression levels reached the peak at 6 h (Figure 6A). Under drought stress, *CsOSCA2.1*, *CsOSCA2.2*, *CsOSCA2.3*, *CsOSCA3.1*, and *CsOSCA4.1* showed significantly up-regulated expression compared with the control, all of which exhibited the highest transcript abundance at 12 h or 24 h (Figure 6B). Notably, *CsOSCA1.1* and *CsOSCA4.1* exhibited the most significant response to salt and drought stress, respectively. Under ABA stress, all tested *CsOSCA* genes displayed similar expression profiles, with a first continuous increase in expression till reaching the peak at 12 h, followed by a decrease at the later time point (24 h) (Figure 6C), indicating that the *CsOSCA* genes are possibly involved in the ABA signal pathway.

### 3.8. Expression Patterns of CsOSCA Genes under Biotic Stress

**Figure 7:**
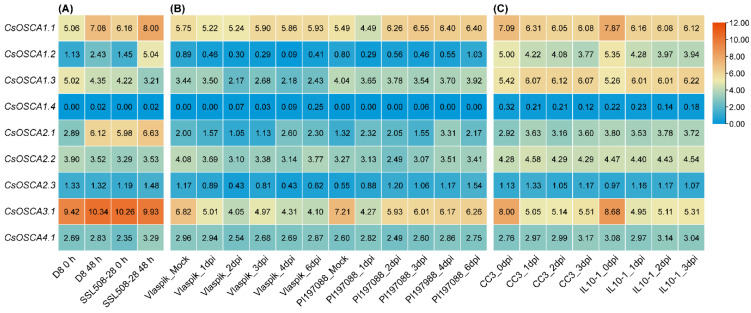
Expression profiles of *CsOSCA* genes under three biotic stress treatments including PM (**A**), DM (**B**), and RKN (**C**). (**A**) Expression analysis of *CsOSCA* genes in D8 (PM susceptible) and SSL508-28 (PM resistant) cucumber plants with and without inoculation of *Sphaerotheca fuliginea*. (**B**) Expression analysis of *CsOSCA* genes in Vlaspik (DM susceptible) and PI 197,088 (DM resistant) cucumber plants with and without inoculation of *Pseudoperonospora cubensis*. (**C**) Expression analysis of *CsOSCA* genes in CC3 (RKN-susceptible) and IL10-1 (RKN resistant) cucumber plants with and without inoculation of *Meloidogyne incognita*. dpi, day post inoculation.

To investigate which *CsOSCA* genes are involved in biotic stress response, we retrieved the TPM values of *CsOSCA* genes under the infection of *S. fuliginea*, *P. cubensis*, and *M. incognita* based on the RNA-seq data, and generated the heatmaps. Under the infection of *S. fuliginea* (PM treatment), the expression levels of three and three *CsOSCA* genes were altered in susceptible and resistant varieties, respectively. Amongst them, *CsOSCA1.1* and *CsOSCA1.2* showed increases in expression in both susceptible and resistant varieties under PM treatment (Figure 7A), suggesting their key roles in PM resistance. In addition, the expression of *CsOSCA1.3* declined in the resistant variety, while *CsOSCA2.1* was up-regulated only in the susceptible variety (Figure 7A), implying that they play different roles in the response to *S. fuliginea* infection. Under the infection of *P. cubensis* (DM treatment), *CsOSCA1.1* and *CsOSCA2.1* were induced in the resistant variety but not in the susceptible variety; *CsOSCA1.3* was down-regulated in the susceptible variety but not in the resistant variety; while *CsOSCA3.1* was significantly down-regulated in both the susceptible and resistant varieties (Figure 7B). Under the infection of *M. incognita* (KRN treatment), three genes (*CsOSCA1.1*, *CsOSCA1.2* and *CsOSCA3.1*) showed significant decreases in expression in both the susceptible and resistant varieties (Figure 7C). These results indicated that the *CsOSCA* genes might play an important role during cucumber disease infection.

## 4. Discussion

Genome-wide surveys and identification of the *OSCA* gene family have been widely performed in different plant species, but the number of *OSCA* genes varies greatly among different species. In this study, a total of nine *OSCA* genes were identified in cucumber genome, and the number was smaller than the 11 in rice [15], 12 in maize [18,24], 12 in tomato [28], 15 in *Arabidopsis* [10], 16 in pear [29], and 21, 22, and 35 in *Gossypium arboreum*, *G. raimondii*, and *G. hirsutum*, respectively [16]. Gene duplication events have played a key role in the expansion of *OSCA* gene family in different plants, such as maize [18,24], pear [29], and *G. hirsutum* [16], resulting in different members of this family in different plants. However, no tandem and segmental duplication events were observed in *CsOSCA* genes (data not shown), which might have eventually contributed to the small number of *OSCA* genes in cucumber. Cucumber OSCA proteins were clustered into four phylogenetic clades with OSCAs from *Arabidopsis*, rice and maize, and clade I and II obviously have more OSCAs than clade III and IV (Figure 1) as reported previously [14,15,28]. In addition, each CsOSCA had at least one homolog from other three species, indicating that the OSCAs in the representing clades may have occurred from common ancestors. Moreover, nearly all *CsOSCAs* had a consistent conserved motif arrangement (Figure 2A). Gene structure analysis revealed that the *CsOSCA* genes had clade-specific exon-intron patterns, and some genes had similar exon/intron structures but different sequence lengths (Figure 2B), which is also the case in rice [15], *G. hirsutum* [16], and potato [30]. The highly conserved evolutionary relationship, conserved motif and exon-intron arrangement of the *OSCA* genes indicated that the members in the same group probably have conserved functions after gene duplication and phylogenetic diversification events [14].

A number of studies have demonstrated the significant functions of *OSCA* genes in response to osmotic-related abiotic stresses. For example, a total of 11 *OsOSCA* genes were identified in rice; ten of them displayed correlation with osmotic alters at different levels [15]. Additionally, *OsOSCA1.1* has been reported to be involved in hyperosmolality and salt stress sensing, which are associated with stomatal closure and seedling survival [31]. In this study, various stress- and hormone-responsive *cis*-elements that play essential roles in stress response through regulation of stress-responsive genes were found in the promoter regions of *CsOSCA* genes (Figure 4), implying the possible roles of *CsOSCA* genes in stress response. Multiple *cis*-elements involved in multiple stress responses (hormones and abiotic stresses) have also been identified in the promoters of *OSCA* genes in other plant species, such as *G. hirsutum* [16], maize [18], soybean [9], tomato [28], and potato [30]. We then examined the transcript levels of seven selected *CsOSCA* genes under three osmotic-related abiotic stresses including drought, salt and ABA by qRT-PCR. Notably, the expression of *CsOSCA2.2*, *CsOSCA3.1* and *CsOSCA4.1* was up-regulated under salt and drought stress (Figure 6A,B), suggesting their positive roles in response to salt and drought stress. Cotton *GhOSCA1.1* is induced by salt and dehydration stress, and *GhOSCA1.1* virus-induced gene silenced plants displayed an increase in salt and dehydration stress sensitivity, revealing that the *GhOSCA1.1* gene plays a positive role in response to salt and drought stress [16]. It is worth noting that all selected *CsOSCA* genes showed significant increases in expression under ABA treatment (Figure 6C), implying that the *CsOSCA* genes are possibly involved in the ABA signal pathway. Similar results were also observed in other plants. For example, 12 out of 13 *Vigna radiata VrOSCA* genes showed increases in expression under NaCl, PEG and ABA treatments [32]. Of the 12 *OSCA* genes in maize, 11 *ZmOSCA* genes were up-regulated in response to NaCl, PEG and ABA treatments [18].

Upon pathogen infection, the cytosolic Ca^2+^ concentration can also be elevated, and thereby trigger downstream responses [33]. Therefore, it can be speculated that OSCAs may also play certain roles in the plant response to pathogen invasion. *Arabidopsis* OSCA1.3 was found to control stomatal closure during immune signaling [11]. In this study, the expression levels of five *CsOSCA* genes were altered in both the resistant and/or susceptible cucumber varieties under the infection of phytopathogens (Figure 7). Notably, *CsOSCA1.1* and *CsOSCA1.2* showed elevated expression in both the susceptible and resistant varieties under PM treatment, while their expression was down-regulated in both the susceptible and resistant varieties under RKN treatment (Figure 7A,C). In addition, *CsOSCA3.1* showed down-regulated expression in both the susceptible and resistant varieties under DM and RKN treatments (Figure 7B,C). These findings indicate that these CsOSCAs may act as regulators with roles in cucumber responses to pathogens invasion.

## 5. Conclusions

Overall, nine *OSCA* family members were identified in the cucumber genome with quite conserved motifs, which are distributed on six out of the seven cucumber chromosomes. The cucumber *OSCA* family members were characterized from the perspectives of evolutionary relationships, gene structures, promoter analysis and tissue-expression patterns, which may be associated with their biological roles. Furthermore, the RNA-sequence and qRT-PCR based expression analysis showed that several *CsOSCA* genes had remarkable changes in expression under diverse phytopathogen infection and osmotic-related abiotic stresses, indicating that they play key roles in response to various stresses of cucumbers. This is the first genome-wide survey of *OSCA* genes in cucumber, which provides a solid foundation for future research on the molecular mechanisms of *CsOSCA* genes in stress response, and ultimately assists the development of stress-resistant cucumber varieties.

## Figures and Tables

**Table 1:** Identification and characterization of *OSCA* gene family in cucumber.

Gene	Accession No. (v2)	Chromosome: Position	gDNA/bp	CDS/bp	Protein			
					AA	MW/kDa	pI	GRAVY
*CsOSCA1.1*	Csa1G701320.1	Chr1: 28,326,385–28,336,117	9733	2322	773	87.97	9.16	0.186
*CsOSCA1.2*	Csa2G416110.1	Chr2: 21,427,024–21,432,896	5873	2307	768	87.75	8.93	0.168
*CsOSCA1.3*	Csa3G006670.1	Chr3: 814,253–819,247	4995	2388	795	91.15	9.32	0.134
*CsOSCA1.4*	Csa7G392390.1	Chr7: 14,860,224–14,868,316	8093	2271	756	86.89	8.78	0.156
*CsOSCA2.1*	Csa2G352960.1	Chr2: 16,330,484–16,335,086	4603	2232	743	84.16	8.97	0.261
*CsOSCA2.2*	Csa5G606420.1	Chr5: 22,846,868–22,853,705	6838	2139	712	80.73	8.81	0.364
*CsOSCA2.3*	Csa5G623630.1	Chr5: 24,915,913–24,922,103	6191	2136	771	80.66	8.63	0.269
*CsOSCA3.1*	Csa6G525220.1	Chr6: 28,314,457–28,318,525	4069	2178	725	81.87	9.37	0.310
*CsOSCA4.1*	Csa6G507280.1	Chr6: 25,978,314–25,981,392	3079	2430	809	92.01	6.42	0.178

## Data Availability

The data presented in this study are available within the article and Supplementary Materials.

## Supplementary Materials

The following supporting information can be downloaded at: www.mdpi.com/article/10.3390/biology11081134/s1, Table S1: The gene-specific primers used for qRT-PCR.

## References

[1] Garrido-Gala J., Higuera J.J., Muñoz-Blanco J., Amil-Ruiz F., Caballero J.L. (2019). The VQ motif-containing proteins in the diploid and octoploid strawberry. Sci. Rep..

[2] Zhu C., Xiao L., Hu Y., Liu L., Liu H., Hu Z., Liu S., Zhou Y. (2022). Genome-wide survey and expression analysis of B-box family genes in cucumber reveal their potential roles in response to diverse abiotic and biotic stresses. Agriculture.

[3] Knight H., Trewavas A.J., Knight M.R. (1997). Calcium signalling in *Arabidopsis thaliana* responding to drought and salinity. Plant J..

[4] Kleist T.J., Wudick M.M. (2022). Shaping up: Recent advances in the study of plant calcium channels. Curr. Opin. Cell Biol..

[5] Jammes F., Hu H.C., Villiers F., Bouten R., Kwak J.M. (2011). Calcium-permeable channels in plant cells. FEBS J..

[6] Swarbreck S.M., Colaço R., Davies J.M. (2013). Plant calcium-permeable channels. Plant Physiol..

[7] Davies J.M. (2014). Annexin-mediated calcium signalling in plants. Plants.

[8] Nawaz Z., Kakar K.U., Ullah R., Yu S., Zhang J., Shu Q.Y., Ren X.L. (2019). Genome-wide identification, evolution and expression analysis of cyclic nucleotide-gated channels in tobacco (*Nicotiana tabacum* L.). Genomics.

[9] Zeng H., Zhao B., Wu H., Zhu Y., Chen H. (2020). Comprehensive in silico characterization and expression profiling of nine gene families associated with calcium transport in soybean. Agronomy.

[10] Yuan F., Yang H., Xue Y., Kong D., Ye R., Li C., Zhang J., Theprungsirikul L., Shrift T., Krichilsky B. (2014). OSCA1 mediates osmotic-stress-evoked Ca^2+^ increases vital for osmosensing in *Arabidopsis*. Nature.

[11] Thor K., Jiang S., Michard E., George J., Scherzer S., Huang S., Dindas J., Derbyshire P., Leitão N., DeFalco T.A. (2020). The calcium-permeable channel OSCA1.3 regulates plant stomatal immunity. Nature.

[12] Liu X., Wang J., Sun L. (2018). Structure of the hyperosmolality-gated calcium-permeable channel OSCA1.2. Nat. Commun..

[13] Zhang M., Wang D., Kang Y., Wu J.X., Yao F., Pan C., Yan Z., Song C., Chen L. (2018). Structure of the mechanosensitive OSCA channels. Nat. Struct. Mol. Biol..

[14] Wu X., Yuan F., Wang X., Zhu S., Pei Z.M. (2022). Evolution of osmosensing OSCA1 Ca^2+^ channel family coincident with plant transition from water to land. Plant. Genome.

[15] Li Y., Yuan F., Wen Z., Li Y., Wang F., Zhu T., Zhuo W., Jin X., Wang Y., Zhao H. (2015). Genome-wide survey and expression analysis of the *OSCA* gene family in rice. BMC Plant Biol..

[16] Yang X., Xu Y., Yang F., Magwanga R.O., Cai X., Wang X., Wang Y., Hou Y., Wang K., Liu F. (2019). Genome-wide identification of OSCA gene family and their potential function in the regulation of dehydration and salt stress in *Gossypium hirsutum*. J. Cotton Res..

[17] Tong K., Wu X., He L., Qiu S., Liu S., Cai L., Rao S., Chen J. (2022). Genome-wide identification and expression profile of *OSCA* gene family members in *Triticum aestivum* L.. Int. J. Mol. Sci..

[18] Cao L., Zhang P., Lu X., Wang G., Wang Z., Zhang Q., Zhang X., Wei X., Mei F., Wei L. (2020). Systematic analysis of the maize *OSCA* Genes revealing *ZmOSCA* family members involved in osmotic stress and ZmOSCA2.4 confers enhanced drought tolerance in transgenic *Arabidopsis*. Int. J. Mol. Sci..

[19] Zhai Y., Wen Z., Fang W., Wang Y., Xi C., Liu J., Zhao H., Wang Y., Han S. (2021). Functional analysis of rice *OSCA* genes overexpressed in the *Arabidopsis osca1* mutant due to drought and salt stresses. Transgenic Res..

[20] Zhai Y., Wen Z., Han Y., Zhuo W., Wang F., Xi C., Liu J., Gao P., Zhao H., Wang Y. (2020). Heterogeneous expression of plasma-membrane-localised OsOSCA1.4 complements osmotic sensing based on hyperosmolality and salt stress in *Arabidopsis osca1* mutant. Cell Calcium.

[21] Akita K., Miyazawa Y. (2022). The mechanosensitive Ca^2+^ channel, OSCA1.1, modulates root hydrotropic bending in *Arabidopsis thaliana*. Environ. Exp. Bot..

[22] Liao L., Hu Z., Liu S., Yang Y., Zhou Y. (2021). Characterization of germin-like proteins (GLPs) and their expression in response to abiotic and biotic stresses in cucumber. Horticulturae.

[23] Liu X., Lu H., Liu P., Miao H., Bai Y., Gu X., Zhang S. (2020). Identification of novel loci and candidate genes for cucumber downy mildew resistance using GWAS. Plants.

[24] Ding S., Feng X., Du H., Wang H. (2019). Genome-wide analysis of maize *OSCA* family members and their involvement in drought stress. PeerJ.

[25] Hu B., Jin J., Guo A.Y., Zhang H., Luo J., Gao G. (2015). GSDS 2.0: An upgraded gene feature visualization server. Bioinformatics.

[26] Chen C., Chen H., Zhang Y., Thomas H.R., Frank M.H., He Y., Xia R. (2020). TBtools: An integrative toolkit developed for interactive analyses of big biological data. Mol. Plant.

[27] Li Z., Zhang Z., Yan P., Huang S., Fei Z., Lin K. (2011). RNA-Seq improves annotation of protein-coding genes in the cucumber genome. BMC Genom..

[28] Waseem M., Aslam M.M., Shaheen I. (2021). The DUF221 domain-containing (DDP) genes identification and expression analysis in tomato under abiotic and phytohormone stress. GM Croops Food.

[29] Gu X., Wang P., Liu Z., Wang L., Huang Z., Zhang S., Wu J. (2018). Genome-wide identification and expression analysis of the *OSCA* gene family in *Pyrus bretschneideri*. Can. J. Plant Sci..

[30] Zaynab M., Peng J., Sharif Y., Albaqami M., Al-Yahyai R., Fatima M., Nadeem M.A., Khan K.A., Alotaibi S.S., Alaraidh I.A. (2022). Genome-wide identification and expression profiling of DUF221 gene family provides new insights into abiotic stress responses in potato. Front. Plant Sci..

[31] Han Y., Wang Y., Zhai Y., Wen Z., Liu J., Xi C., Zhao H., Wang Y., Han S. (2022). OsOSCA1.1 mediates hyperosmolality and salt stress sensing in *Oryza sativa*. Biology.

[32] Yin L., Zhang M., Wu R., Chen X., Liu F., Xing B. (2021). Genome-wide analysis of OSCA gene family members in *Vigna radiata* and their involvement in the osmotic response. BMC Plant Biol..

[33] Moeder W., Phan V., Yoshioka K. (2019). Ca^2+^ to the rescue—Ca^2+^ channels and signaling in plant immunity. Plant Sci..

